# Supplementary material for: Predictive Modeling of Phenotypic Antimicrobial Susceptibility of Selected Beta-Lactam Antimicrobials from Beta-Lactamase Resistance Genes
Source: Antibiotics (Basel). 2024 Feb 28;13(3):224. doi: 10.3390/antibiotics13030224 (PMC10967287; doi:10.3390/antibiotics13030224)
Supplement: Supplementary file 1 [file antibiotics-13-00224-s001.zip › antibiotics-2874939-supplementary.pdf]

Supplementary Table S1: Prediction of MIC of Ceftriaxone from retail meat

**Variables**	**Categories**	**coefficients**	**Predicted MIC**	**95% CI**	**P value**
Year	2002	ref			
	2003	0.06	1.04	0.96-1.12	0.32
	2004	0.11	1.08	1.01-1.16	0.03
	2005	0.08	1.06	0.98-1.13	0.13
	2006	0.02	1.01	0.95-1.09	0.67
	2007	0.06	1.04	0.97-1.12	0.27
	2008	0.01	1.01	0.94-1.08	0.81
	2009	0.14	1.10	1.03-1.18	0.01
	2010	0.58	1.49	1.38-1.61	<0.01
	2011	0.01	1.01	0.94-1.08	0.78
	2012	0.00	1.00	0.93-1.07	0.93
	2013	0.00	1.00	0.93-1.07	0.95
	2014	0.00	1.00	0.93-1.08	0.93
	2015	-0.01	1.00	0.93-1.06	0.91
	2016	0.03	1.02	0.96-1.10	0.48
	2017	0.03	1.02	0.96-1.09	0.51
	2018	0.06	1.04	0.98-1.11	0.19
bla$_{\text{CMY-2}}$	(Yes)	5.85	57.59	19.20-172.73	<0.01
bla$_{\text{CMY-3}}$	(Yes)	-0.22	0.86	0.50-1.47	0.58
bla$_{\text{CTX-M-1}}$	(Yes)	7.80	223.23	31.35-1589.55	<0.01
bla$_{\text{CTX-M-55}}$	(Yes)	7.99	254.64	1.90-34080.37	0.03
bla$_{\text{CTX-M-65}}$	(Yes)	7.67	203.68	111.36-372.51	<0.01
bla$_{\text{SHV-2}}$	(Yes)	4.99	31.79	1.16-870.20	0.04
bla$_{\text{TEM-1}}$	(Yes)	0.02	1.01	0.99-1.04	0.28
Bacteria	*E. coli*	ref			
	S. enterica	0.00	1.00	0.97-1.03	0.91
Meat type	Chicken breast	ref			
	Ground Beef	-0.03	0.98	0.94-1.02	0.28
	Ground Turkey	-0.03	0.98	0.96-1.00	0.02
	Pork cut	-0.04	0.97	0.94-1.00	0.08
Interaction	*bla*$_{\text{CMY-2}}$ ×2003	0.58	1.50	0.43-5.25	0.53
	bla$_{\text{CMY-2}}$ ×2004	0.41	1.33	0.42-4.25	0.63
	bla$_{\text{CMY-2}}$ ×2005	0.28	1.21	0.39-3.81	0.74
	bla$_{\text{CMY-2}}$ ×2006	0.45	1.37	0.42-4.44	0.60
	bla$_{\text{CMY-2}}$ ×2007	0.48	1.39	0.40-4.89	0.61
	bla$_{\text{CMY-2}}$ ×2008	0.12	1.09	0.36-3.25	0.88
	bla$_{\text{CMY-2}}$ ×2009	0.07	1.05	0.37-2.99	0.92
	bla$_{\text{CMY-2}}$ ×2010	-0.45	0.73	0.24-2.18	0.57
	bla$_{\text{CMY-2}}$ ×2011	0.13	1.09	0.33-3.58	0.89
	bla$_{\text{CMY-2}}$ ×2012	0.00	1.00	0.31-3.23	1.00
	bla$_{\text{CMY-2}}$ ×2013	-0.22	0.86	0.26-2.88	0.81
	bla$_{\text{CMY-2}}$ ×2014	-0.07	0.95	0.27-3.38	0.94

	bla$_{CMY-2}$ ×2015	-0.18	0.88	0.25-3.09	0.84
	bla$_{CMY-2}$ ×2016	-0.27	0.83	0.24-2.86	0.77
	bla$_{CMY-2}$ ×2017	-0.30	0.81	0.23-2.84	0.74
	bla$_{CMY-2}$ ×2018	-0.12	0.92	0.25-3.36	0.90

The interaction between two variables is represented by (×).

Supplementary Table S2: Prediction of MIC of Cefoxitin from retail meat

**Variables**	**Categories**	**coefficients**	**Predicted MIC**	**95% CI**	**P value**
Year	2002	ref			
	2003	-0.06	0.96	0.85-1.08	0.51
	2004	-0.12	0.92	0.83-1.03	0.16
	2005	-0.50	0.71	0.63-0.79	<0.01
	2006	-0.09	0.94	0.84-1.05	0.28
	2007	-0.17	0.89	0.79-0.99	0.03
	2008	-0.17	0.89	0.80-0.99	0.03
	2009	-0.06	0.96	0.86-1.07	0.48
	2010	-0.02	0.99	0.89-1.10	0.81
	2011	-0.16	0.89	0.80-1.00	0.05
	2012	-0.24	0.85	0.76-0.95	<0.01
	2013	-0.20	0.87	0.78-0.97	0.01
	2014	0.02	1.02	0.91-1.14	0.78
	2015	0.14	1.10	0.99-1.22	0.07
	2016	0.00	1.00	0.90-1.11	0.95
	2017	0.03	1.02	0.92-1.12	0.70
	2018	0.00	1.00	0.91-1.10	1.00
bla$_{CMY-2}$	(Yes)	2.33	5.04	3.97-6.41	<0.01
bla$_{CMY-3}$	(Yes)	-0.15	0.90	0.84-0.96	<0.01
bla$_{CTX-M-1}$	(Yes)	0.34	1.26	0.85-1.87	0.25
bla$_{CTX-M-55}$	(Yes)	1.12	2.17	0.98-4.81	0.06
bla$_{CTX-M-65}$	(Yes)	0.65	1.57	1.38-1.77	<0.01
bla$_{SHV-2}$	(Yes)	0.41	1.33	0.51-3.50	0.56
bla$_{TEM-1}$	(Yes)	0.05	1.04	1.01-1.07	0.02
Bacteria	*E. coli*	ref			
	S. enterica	-0.58	0.67	0.63-0.71	<0.01
Meat type	Chicken breast	ref			
	Ground Beef	-0.17	0.89	0.82-0.96	<0.01
	Ground Turkey	-0.01	0.99	0.93-1.06	0.85
	Pork cut	-0.10	0.93	0.86-1.01	0.09
Interaction	*bla*$_{CMY-2}$ ×2003	0.04	1.03	0.81-1.31	0.81
	bla$_{CMY-2}$ ×2004	1.10	2.14	1.73-2.65	<0.01
	bla$_{CMY-2}$ ×2005	1.28	2.42	1.95-3.01	<0.01
	bla$_{CMY-2}$ ×2006	1.04	2.06	1.66-2.55	<0.01
	bla$_{CMY-2}$ ×2007	0.95	1.93	1.54-2.41	<0.01
	bla$_{CMY-2}$ ×2008	0.91	1.88	1.53-2.32	<0.01

	bla$_{\text{CMY-2}}$ ×2009	0.83	1.77	1.44-2.18	<0.01
	bla$_{\text{CMY-2}}$ ×2010	0.83	1.78	1.44-2.20	<0.01
	bla$_{\text{CMY-2}}$ ×2011	0.82	1.77	1.42-2.20	<0.01
	bla$_{\text{CMY-2}}$ ×2012	0.90	1.86	1.49-2.31	<0.01
	bla$_{\text{CMY-2}}$ ×2013	0.94	1.92	1.54-2.39	<0.01
	bla$_{\text{CMY-2}}$ ×2014	0.72	1.65	1.32-2.07	<0.01
	bla$_{\text{CMY-2}}$ ×2015	0.63	1.55	1.24-1.93	<0.01
	bla$_{\text{CMY-2}}$ ×2016	0.83	1.77	1.42-2.21	<0.01
	bla$_{\text{CMY-2}}$ ×2017	0.75	1.68	1.34-2.10	<0.01
	bla$_{\text{CMY-2}}$ ×2018	0.70	1.62	1.29-2.04	<0.01
	bla$_{\text{CMY-2}}$ × *S. enterica*	0.42	1.34	1.18-1.52	<0.01
	bla$_{\text{CMY-2}}$ × *Ground beef*	-0.21	0.86	0.76-0.98	0.03
	bla$_{\text{CMY-2}}$ × *Ground turkey*	-0.14	0.91	0.86-0.96	<0.01
	bla$_{\text{CMY-2}}$ × *Pork cut*	-0.42	0.75	0.63-0.88	<0.01
	S. enterica × *Ground beef*	0.42	1.34	1.19-1.50	<0.01
	S. enterica × *Ground turkey*	0.18	1.13	1.06-1.22	<0.01
	S. enterica × *Pork cut*	0.42	1.33	1.20-1.48	<0.01

The interaction between two variables is represented by (×).

Supplementary Table S3: Prediction of MIC of Ceftiofur from retail meat

**Variables**	**Categories**	**coefficients**	**Predicted MIC**	**95% CI**	**P value**
Year	2002	ref			
	2003	0.16	1.12	0.98-1.27	0.10
	2004	0.17	1.13	1.00-1.27	0.05
	2005	0.08	1.05	0.94-1.19	0.38
	2006	0.56	1.47	1.31-1.65	<0.01
	2007	0.51	1.43	1.27-1.60	<0.01
	2008	0.57	1.49	1.331.66	<0.01
	2009	0.56	1.47	1.31-1.65	<0.01
	2010	0.67	1.59	1.42-1.78	<0.01
	2011	0.43	1.35	1.20-1.52	<0.01
	2012	0.25	1.19	1.05-1.34	<0.01
	2013	0.40	1.32	1.17-1.48	<0.01
	2014	0.54	1.45	1.29-1.64	<0.01
	2015	0.49	1.40	1.26-1.57	<0.01
bla$_{\text{CMY-2}}$	(Yes)	3.85	14.46	12.76-16.39	<0.01
bla$_{\text{CMY-3}}$	(Yes)	-0.09	0.94	0.91-0.97	<0.01
bla$_{\text{CTX-M-1}}$	(Yes)	3.14	8.82	7.36-10.57	<0.01
bla$_{\text{CTX-M-65}}$	(Yes)	3.19	9.11	7.04-11.79	<0.01
bla$_{\text{SHV-2}}$	(Yes)	3.03	8.18	6.28-10.67	<0.01
bla$_{\text{TEM-1}}$	(Yes)	0.06	1.04	1.02-1.07	<0.01
Bacteria	*E. coli*	ref			

	S. enterica	1.19	2.28	1.18-4.40	0.01
Meat type	Chicken breast	ref			
	Ground Beef	0.14	1.10	1.06-1.14	0.01
	Ground Turkey	0.16	1.11	1.02-1.22	<0.01
	Pork cut	0.20	1.15	1.071.24	<0.01
Interaction	bla_{CMY-2} × 2003	-0.20	0.87	0.75-1.02	0.08
	bla_{CMY-2} × 2004	-0.22	0.86	0.74-0.99	0.04
	bla_{CMY-2} × 2005	-0.28	0.82	0.71-0.95	0.01
	bla_{CMY-2} × 2006	-0.61	0.65	0.57-0.75	<0.01
	bla_{CMY-2} × 2007	-0.67	0.63	0.54-0.73	<0.01
	bla_{CMY-2} × 2008	-0.76	0.59	0.52-0.67	<0.01
	bla_{CMY-2} × 2009	-0.70	0.62	0.54-0.70	<0.01
	bla_{CMY-2} × 2010	-0.77	0.58	0.51-0.67	<0.01
	bla_{CMY-2} × 2011	-0.58	0.67	0.58-0.77	<0.01
	bla_{CMY-2} × 2012	-0.40	0.76	0.66-0.87	<0.01
	bla_{CMY-2} × 2013	-0.54	0.69	0.60-0.79	<0.01
	bla_{CMY-2} × 2014	-0.68	0.62	0.54-0.72	<0.01
	bla_{CMY-2} × 2015	-0.64	0.64	0.56-0.74	<0.01
	bla_{CMY-2} × *Ground beef*	-0.17	0.89	0.85-0.93	0.01
	bla_{CMY-2} × *Ground turkey*	-0.21	0.86	0.78-0.96	<0.01
	bla_{CMY-2} × *Pork cut*	-0.59	0.67	0.56-0.79	<0.01

The interaction between two variables is represented by (×).
